# Are We Meeting the Needs? A Systematic Review of Nutritional Gaps and Growth Outcomes in Children with Multiple Food Allergies

**DOI:** 10.3390/nu17091590

**Published:** 2025-05-05

**Authors:** Gianluca Di Cesare, Annalisa Carciofi, Francesca Borgiani, Deborah Cappelletti, Alessio Correani, Chiara Monachesi, Simona Gatti, Maria Elena Lionetti

**Affiliations:** 1 Department of Pediatrics, Polytechnic University of Marche, 60123 Ancona, Italy; gianluca.dicesare@ospedaliriuniti.marche.it (G.D.C.); annalisa.carciofi@ospedaliriuniti.marche.it (A.C.); francesca.borgiani@ospedaliriuniti.marche.it (F.B.); s.gatti@staff.univpm.it (S.G.); m.e.lionetti@staff.univpm.it (M.E.L.); 2 Department of Pediatrics and Neonatology, Provincial General Hospital, 62100 Macerata, Italy; deborah.cappelletti@sanita.marche.it; 3 Department of Odontostomatologic and Specialized Clinical Sciences, Polytechnic University of Marche, 60131 Ancona, Italy; a.correani@univpm.it

**Keywords:** food hypersensitivity, growth impairment, nutrients, dietary counseling

## Abstract

Background/Objectives: Food allergies represent a growing public health challenge, showing an alarming increase in prevalence over the past few decades. Children with multiple food allergies face not only allergic reaction risks but also nutritional gaps, affecting diet, nutrition, and growth. This review summarizes the impact on nutrient intake and growth, highlighting key challenges and strategies to improve clinical care. Methods: The literature search was conducted using a structured search strategy in PubMed up to 31 January, using MeSH terms with Boolean operators (AND, OR) to combine searches (food hypersensitivity AND growth, food hypersensitivity AND nutrition, food hypersensitivity AND micronutrient, food hypersensitivity AND vitamin, food hypersensitivity AND trace element, and soy hypersensitivity) for human studies meeting pre-defined PICOS criteria that simultaneously assessed the reproducibility and validity. Results: Nearly 2000 studies were identified in the literature search, with 31 articles selected for full-text evaluation and 11 articles included in the final review. Dietary restrictions imposed by the need to avoid multiple allergens can lead to a reduced intake of essential nutrients, particularly proteins, calcium, iron, zinc, iodine, folate, vitamin B12, and vitamin D. Children with multiple FAs appear to be at an increased risk of impaired growth, as evidenced by the lower height-for-age, weight-for-age, head-circumference, and weight-for-length Z-scores compared to non-allergic peers. Conclusions: Evidence from the studies reviewed suggests that children with multiple FAs may be at increased risk for growth impairments and nutritional inadequacies, especially where dietary management does not adequately compensate for nutrient losses, and highlights that tailored dietary counseling is crucial.

## 1. Introduction

Food allergies (FAs) showed an alarming increase in prevalence over the past few decades, affecting up to 9% of children worldwide, with a significant variation in FA burden attributed to a complex interplay of genetic, socio-economic, and environmental factors, thus representing a growing public health challenge [1].

The major food allergens, often referred to as the “Big 8”, include peanuts, tree nuts (such as almonds, walnuts, cashews, hazelnuts, pistachios, and Brazil nuts), milk (cow’s milk), eggs (both egg whites and yolks), fish (such as salmon, tuna, and cod), shellfish (including shrimp, lobster, and crab), wheat, and soy, accounting for over 90% of FAs. In the European context, Regulation (EU) No. 1169/2011 mandates the labeling of 14 specific food allergens, which include but are not limited to the “Big 8”. This regulatory framework plays a crucial role in guiding elimination diets and informing consumers, particularly those managing multiple food allergies [2]. In addition to the most common food allergens, more than 170 other foods have been identified as potential triggers for allergic reactions. These allergens include a diverse range of foods, such as fruits, vegetables, seeds, legumes, and spices, many of which are commonly consumed as part of a balanced diet. For instance, sesame seeds, mustard, buckwheat, and lupin are not only potential allergens but also important sources of nutrients, including healthy fats and proteins [3].

The cornerstone of FA management remains the strict elimination of offending allergens. Organizations such as the Codex Alimentarius, under the joint FAO/WHO initiative, play a key role in setting international standards for food safety, including the identification of priority allergens. Codex has outlined guidelines for allergen labeling and classification, which are intended to help consumers make informed choices, particularly in countries with diverse diets. Similarly, the European Food Safety Authority (EFSA) supports the identification of food allergens and their regulation within Europe, offering scientific advice on allergen risk assessment. Both EFSA and Codex Alimentarius recognize that FAs extend beyond the eight most common food allergens and encourage more comprehensive efforts to manage food safety and protect public health. As FAs continue to rise globally, these organizations remain central in developing strategies that address both immediate allergic risks and the longer-term nutritional challenges faced by affected individuals, particularly children.

However, for children diagnosed with multiple FAs, the impact extends beyond the immediate risk of allergic reactions, and the avoidance of these foods can result in nutritional gaps, profoundly influencing their dietary patterns [4].

Furthermore, the dietary restriction of nutrient-rich foods often results in challenges that go beyond the kitchen table. Studies have highlighted that children with FAs are at increased risk of developing avoidant behaviors toward food, nutrient deficiencies, such as calcium, vitamin D, and essential fatty acids, as well as potential disruptions in growth and development [5].

Despite advances in nutritional science and the availability of hypoallergenic formulas offering promising solutions to mitigate these risks, the interplay between allergic inflammation, nutrient metabolism, and psychosocial factors still complicates the clinical management of these children.

The aim of this systematic review is to present an updated summary of the nutritional impact of pediatric FAs in terms of 1. nutritional adequacy (dietary intake and nutritional deficiencies) and 2. growth outcomes in order to identify key challenges and potential strategies for optimizing clinical care in this vulnerable population.

## 2. Materials and Methods

### 2.1. Search Strategy

Full-text articles written in English, indexed in PubMed, and published from January 1990 up to January 2025 were considered for inclusion, while studies available only as abstracts were excluded. The following MeSH terms with Boolean operators (AND, OR) to combine searches were used to identify eligible articles: food hypersensitivity AND growth, food hypersensitivity AND nutrition, food hypersensitivity AND micronutrient, food hypersensitivity AND vitamin, food hypersensitivity AND trace element, and soy hypersensitivity. The search strings were developed collaboratively by the authors. References of the selected studies were screened to identify other relevant articles.

### 2.2. Inclusion and Exclusion Criteria

Strict inclusion and exclusion criteria were applied to ensure relevance and quality.

#### 2.2.1. Inclusion Criteria

Children aged 0–18 years.

IgE-mediated FAs confirmed by skin prick tests, serum-specific IgE levels, or food challenge tests.

Studies on children with two or more FAs.

Randomized controlled trials, observational studies, and case reports published in English from January 1990 to January 2025.

#### 2.2.2. Exclusion Criteria

Non-peer-reviewed articles, reviews, conference abstracts, commentaries, and letters to the editor.

No full text available.

Articles published in languages other than English.

#### 2.2.3. Selection Process

The review was conducted between November 2024 and January 2025. Records were screened independently by two authors to ensure objectivity. Disagreements were resolved through discussion and consensus of all authors. The authors achieved a 100% agreement on the inclusion and exclusion of articles after a discussion in which individual articles were evaluated according to the inclusion criteria.

All articles met the minimum criterion of regularity in the procedures, as defined by the PICOS strategy [6], and therefore were included in the analysis. The methodology was developed in accordance with the protocol for reporting systematic reviews proposed by the Preferred Reporting Items for Systematic Reviews and Meta-Analysis (PRISMA) (http://www.prisma-statement.org).

The methodological quality of the included studies was evaluated using the Joanna Briggs Institute (JBI) Critical Appraisal Tool. The key aspects assessed included the appropriateness of participant selection, the clarity and reliability of outcome measurements, the management of potential confounding factors, as well as the robustness of study design and statistical analysis. Based on these criteria, each study was categorized as having a high, moderate, or low risk of bias. All included studies met the quality criteria and were rated as having a low risk of bias.

## 3. Results

**Figure 1:**
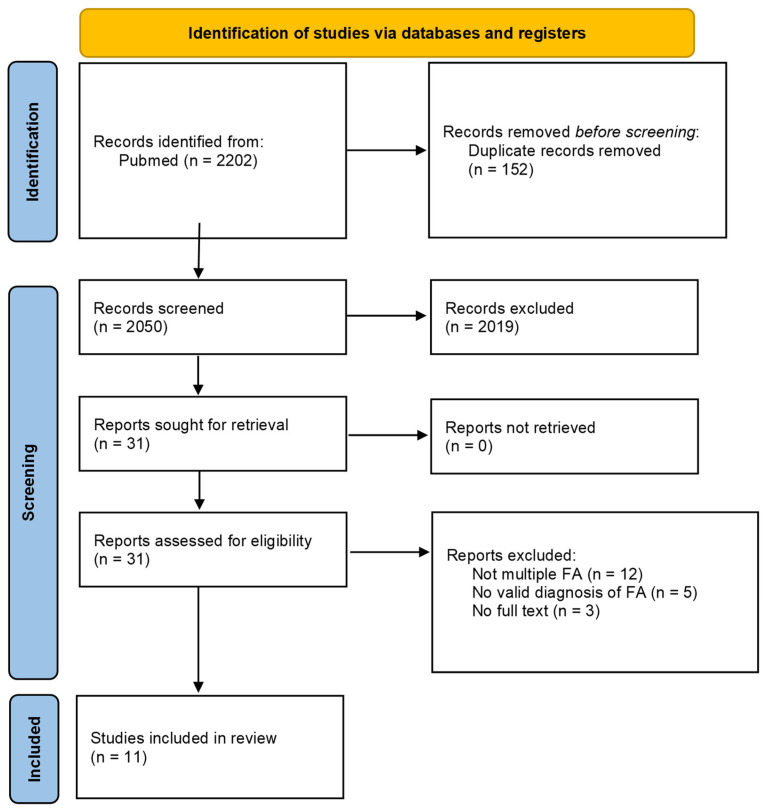
PRISMA flow diagram of the search results from the databases.

Nearly 2000 studies were identified in the literature search. Figure 1 reports the PRISMA diagram illustrating the selection process. After a review of titles and abstracts and the removal of duplicates, 31 articles were considered for full-text evaluation. Full-text reading greatly reduced the number of articles to be critically analyzed to 11. The main findings of the selected studies are summarized in Table 1.

The present review included six cross-sectional studies, two non-controlled trials, one case series, one non-randomized, prospective interventional study, and one retrospective chart review. In total, all the studies examined 1365 food-allergic children and 9968 healthy controls. Cow’s milk allergy (CMA) was the most frequently cited food allergy, appearing in 9 studies, followed by egg allergy in 7 studies and peanut allergy in 5 studies. Other reported food allergies included soy, wheat, tree nuts, fish, shellfish, and sesame.

### 3.1. Impact of Multiple Pediatric FAs in Nutritional Intake and Nutrient Deficiencies

The analysis of the selected articles highlights a significant impact of multiple FAs on dietary intake in affected children. One of four studies reported that children with multiple FAs exhibit lower energy intake compared to healthy peers [7]. However, energy intake was compared to that of healthy controls, but not explicitly assessed against age-specific dietary reference values, limiting the ability to evaluate its adequacy in relation to established nutritional guidelines.

One study [7] analyzed longitudinally the dietary intake and found a significant improvement in the nutritional composition of the diet after a dietic follow-up.

Several studies reported a reduced intake of essential nutrients, with proteins being the most frequently mentioned (in 4 out of 6 studies), with protein deficiency identified in 18% of FA patients at enrollment, as noted by Berni-Canani [7].

**Table 1:** Overview of studies (n = 11).

Study, Design, Class/Rating	Subjects	Comparison Group	Relationship of Multiple FAs to Growth (Standard Used)	Relationship of Multiple FAs to Nutrient Intake (Nutrition Assessment Method/Standard Used)	Limitations and Comments
[8] Christie et al. (2002), cross-sectional	consecutive patients with ≥1 FA * (n = 98) age 3.7 ± 2.3 years * FA: peanut, egg, and cow’s milk	age-matched controls without FA (n = 99) age 4.1 ± 2.4 years	(CDC growth charts) ≥2 FAs more likely to be <25th percentile height-for-age than 1 FA (35% and 16%, respectively) 1 FA more likely to be >75th percentile height-for-age than ≥2 FAs ^c^ (34% and 13%, respectively)	(3-day food record/10th edition DRIs) ≥1 FA more likely to consume >100% DRI for vitamin E than controls (78% and 92%, respectively) milk allergy more likely to consume <100% DRI for calcium than other allergies (58% and 31%, respectively) other allergies more likely to consume <100% DRI vitamin E than milk allergy (90% and 62%, respectively) FA without nutrition counseling more likely to consume< 67% DRI for calcium and vitamin D than those with counseling (calcium: 39% and 15%, respectively; vitamin D: 55% and 29%, respectively)	used 25th percentile height–forage as indicator of potential undernutrition did not report weight data unknown time avoiding allergens
[9] Flammarion et al. (2011), cross-sectional	children with ≥1 FA * (n = 96) age 4.6 ± 2.58 years * FA: peanut, cow’s milk, soy, and fish	age- and sex- matched controls (n = 95) age 4.7 ± 2.7 years	(Sempé reference values) ≥3 FAs more likely to have weight–for-age and height-for-age z-score < −2 than children with 1–2 FAs (weight: 14.5% and 1.8%, respectively; height: 12.1% and 3.6%, respectively) FA more likely than controls to have weight-for-age and height-for-age z-scores < −2 (weight: 9.3% and 0%, respectively; height: 7.2% and 2.7%, respectively)	(3-day food record/RDI for healthy children in France) FA consumed more vitamins A and E than controls (vitamin A: 95% RDA and 65% RDA, respectively; vitamin E: 92% RDA and 53% RDA, respectively)	88% of children with food allergies received nutrition counseling by a dietitian avoiding allergens for ≥4 months prior to study
[10] Cho et al. (2011), case series	children with atopic dermatitis and ≥1 FA * (n = 112) classified according to sensitized FA * age 16.00 ± 10.93 months * FA: egg white, cow’s milk, soy, peanut, wheat, and fish	children with atopic dermatitis and no FA (n = 53)	(Korean growth charts) weight-for-age z-score decreased as sensitized FA increased (−0.21 ± 1.08, 0.02 ± 0.87, −0.14 ± 0.96, and −0.57 ± 1.90 for 0, 1, 2, and ≥3 sensitized FA, respectively) ≥3 FAs significantly shorter than 1 FA (−0.50 ± 1.01 and 1.10 ± 0.90, respectively)		unknown time avoiding allergens did not state if they were actually avoiding the sensitized FA
[11] Aldámiz-Echevarría et al (2008), cross-sectional	children with ≥2 FAs * (n = 25) age 3.8 ± 1.6 years * FA: egg, cow’s milk, fish, legumes, meat, cereals, fruits, vegetables, molluscs, and crustaceans	healthy children (n = 61) age 5.1 ± 2.6 years	WNL	(no nutrition assessment measure used/no nutrient intake standard used) EFA index [(ω3 + ω6)/(ω7 + ω9)] was lower in FA than controls (1.5 and 1.9, respectively) DHA:DPA ratio was lower in FA than controls (4.0 and 11.7, respectively) lower percentage of α-linoleic acid in FA than controls (27.4 and 30.7, respectively)	unknown time avoiding allergens diets of the control patients were not described
[12] Sicherer et al. (2001), non-controlled trial	children with ≥1 FA * (n = 29) age 6–210 months at enrollment * FA: soy, egg, pea, potato, banana, wheat, barley, beef, peanut, oat, rice, corn, pork, chicken	no FA (n = 2)	(NCHS) weight-for-age, height-for-age, and weight-for-height z-scores WNL at entry, 1 month, 4 months (−0.8 ± 0.2, −0.3 ± 0.2, and −0.6 ± 0.3, respectively, at 4 months) no statistically significant differences in z-scores between 0, 1, and 4 months	(diet record/RDA) consumed 152 ± 12% RDA for protein from formula at 4 months	unknown time avoiding allergens unclear how many had 18 multiple FAs vs. only milk allergy funded by formula manufacturer primary purpose to test efficacy of formula many with allergic eosinophilic gastroenteritis
[13] Zeiger et al. (1999), non-controlled trial	children with ≥1 FA * (n = 93) children with allergy to milk and soy (n = 13) 11 had additional allergies age 23.4 ± 2.5 months at enrollment * FA: egg, peanut, meat, and wheat	children with milk allergy but no soy allergy (n = 80) age 18.4 ± 0.9 months	(NCHS) children with milk and soy allergy had lower height-for-age z-scores than children with milk allergy (−0.73 ± 0.26 vs. −0.09 ± 0.12)		unknown time avoiding allergens purpose of study was to assess prevalence of soy allergy in children with milk allergy weight and height for milk and soy allergic children measured only one time
[14] Papachristou et al. (2024), cross-sectional	children with diagnosed IgE- mediated FA * (n = 100) age 8.5 ± 3.7 children with multiple FAs constitute 50% (n = 50) * FA: nut, cow’s milk, egg, fish, legumes, fruits, sesame, wheat, shellfish, soy	children with respiratory allergy (n = 60) age 10.2 ± 3.6	BMI (International Obesity Task Force). Children with FAs had lower mean BMI compared with controls (17.3 ± 3.2 vs. 19.4 ± 3.9; *p* < 0.001). The FA group presented higher frequency of underweight (19.6% vs. 5.1%), and lower frequencies of overweight (14.4% vs. 25.4%) and obesity (6.2% vs. 10.2%).	dietary intake was assessed by registered dietitians through 4 24 h recalls. Children with FAs consumed fewer servings of fermented dairy products (0.9; 95% CI, 0.7, 1.1 vs. 1.2; 95% CI, 0.9, 1.4 servings/day) but more servings of plant-based dairy alternatives (0.1; 95% CI, 0.05, 0.2 vs. 0.0; 95% CI, 0.0, 0.07 servings/day) and meat (1.7, 95% CI, 1.6, 1.9 vs. 1.5, 95% CI, 1.3, 1.7 servings/day) than controls. No difference was observed in the diet diversity between the two groups (11–12 different foods/day). Within the FA group, children with allergy to milk proteins had lower energy intake from protein, lower intake of calcium, lower consumption of commercially prepared sweets, and higher consumption of eggs, compared with children with nut or egg allergy, but no difference in diet diversity was observed.	study that does not allow for the assessment of growth trajectories time since diagnosis was not considered no biomarker was measured the higher percentage of underweight in children with FAs suggests the need for targeted nutrition intervention after FA diagnosis
[15] Dziechciarz P. et al. (2024), cross-sectional, multi-center study	children with confirmed CMA (n = 144, 57% multiple FAs *) and suspected CMA (n = 88, 78% multiple FAs *) 51% of all participants with multiple FAs aged 6–24 months mean duration of CMP-free diet was 6.5 months * FA: cow’s milk		(weight for length and BMI for age were converted into SD scores (z-scores) based on WHO Standards using the WHO Anthro Software) according to weight for length z-score, 36% of all included children (n = 232) had any nutritional impairment (weight for length z-score above 1 or below −1): moderate malnutrition (10%), mild malnutrition (10.3%), possible risk of overweight (11.2%), no significant changes was found between nutritional status in children with confirmed and suspected CMA according to BMI for age z-score, 31.5% of all included children (n = 232) had any nutritional impairment (BMI for age z-score above 1 or below −1): mild malnutrition (9%), possible risk of overweight (9.9%) among patients with confirmed CMA, 30.5% had any nutritional impairment: mild malnutrition (9%), possible risk of overweight (9.7%)		the absence of a control group prevents comparison with a healthy population nutritional status was assessed at only one time point the cross-sectional nature of the survey precludes any causal inferences malnutrition and overweight are a burden in Polish children up to 2 years of age, with suspected and confirmed by oral food challenge CMA on a CMP-free diet
[7] Roberto Berni Canani et al. (2014), non-randomized, prospective, multicenter intervention study	children with FAs * aged 6 to 36 months who were following an elimination diet without dietary counseling for at least 60 days (n = 91) age 18.9 months (95% CI, 16.5 to 21.3) * FA: cow’s milk, hen’s egg, soy, fish, tomato, wheat, legumes, and rice multiple food allergy n = 42 (46.1%)	healthy children without FAs (aged 6 to 36 months) (n = 66) mean age 20.3 months (95% CI 17.7 to 22.8)	anthropometric indexes (z score for weight, length/height, and head circumference) were determined using the Euro-Growth References. A weight-to-length ratio <2 standard deviations was more frequent in children with FAs than in children without FAs (21% vs. 3%; *p* < 0.001).	at enrollment, energy and protein intakes were lower in children with FAs (91 kcal/kg/day, interquartile range [IQR] 15.1, minimum [MIN] 55.2, maximum [MAX] 130.6; and 2.2 g/kg/day, IQR 0.5, MIN 1.5, MAX 2.7, respectively) than in children without FAs (96 kcal/kg/day, IQR 6.1, MIN 83.6, MAX 118.0; and 4.6 g/kg/day, IQR 1.2, MIN 2.0, MAX 6.1, respectively; *p* < 0.001). At 6 months following dietary counseling, the total energy intake of children with FAs was similar to the baseline values of control children	the lack of follow-up data on children without FA and the lack of a control group of children with FA not receiving dietary counseling since suboptimal nutritional status is a frequent problem for children with FAs; dietary counseling, provided by a dietitian, is an effective strategy to promote rapid nutritional recovery in these children
[16] Mehta H. et al. (2014), retrospective chart review	FA * children (n = 439), median age 49 months (range, 0–238 months; *t*-test, *p* < 0.001) commercial insurance (n = 79) vs. state insurance (n = 360) * FA: peanut, tree nut, egg, fish, shell fish, wheat, and cow’s milk allergy	non food allergic children (n = 9499), median age 68 months (range, 0–240 months) commercial insurance (n = 1560) vs. state insurance (n = 7939)	(height and weight z-scores were calculated using the CDC and Prevention 2000 growth curves for children aged 2 and older (using the CDC and Prevention growth chart algorithm for SAS—SAS Version 9.3; SAS Inc., Cary, NC, USA) and WHO growth curves for ages younger than 2 years of age). Of those with commercial insurance, children with food allergies were significantly shorter (mean height z-score = 0.06; *p* = 0.01) and weighed less (mean weight z-score −0.1; *p* = 0.006) than children without food allergies (mean height z-score = 0.42; mean weight z-score = 0.07)	children with food allergies may have easier access to processed foods that may be poor substitutions for the allergen(s) that they are avoiding in regard to nutritional content but may contain a high caloric value there was a significant effect in children aged 2–5 and a trend toward significance in children 6–11 years of age, with children having a milk allergy being smaller as older children may not have an adequate substitution with alternative nutrient sources. Milk substitutes for this older age group are lower in fat and most are very low in protein or protein-free; thus, even when they are used, they provide a poor nutritional substitute.	limitations of this study include the retrospective design, reliance on physician diagnosis of food allergy, and lack of serum IgE, prick skin test, and oral food challenge data comorbidities, medication use, nutritional supplementation (e.g., vitamin D and calcium), visit to a dietitian, education regarding nutrition, and systemic symptoms indicating underlying inflammation were not obtained and can all impact growth
	(identified by the International Classification of Diseases, 9th Revision [ICD-9] code for well child visit [v20.2])	(identified by the International Classification of Diseases, 9th Revision [ICD-9] code for well child visit [v20.2])	children with food allergies and state insurance were not smaller in height or weight compared with children without food allergies among White subjects, there was a significant effect of food allergies on height and weight (ANOVA for height *p* = 0.012, for weight *p* = 0.0036) that was not observed for Hispanic/Latino, Black, or Asian subjects children with allergies to milk weighed significantly less than children without milk allergies (*p* = 0.0006)	the combination of poor milk substitution and the elimination of a variety of foods because of milk content in this age group may be contributing to lower energy and protein intakes	results indicate the importance of closely monitoring the growth of children with food allergies, particularly those avoiding milk nutritional counseling should be provided to the families of these children to better educate caregivers on appropriate nutritionally dense substitutes
[17] K. Maslin et al. (2018) Cross-sectional study	presence of a confirmed diagnosis of IgE or non-IgE mediated allergy to at least	healthy participants, who consumed an unrestricted diet	there was no difference in BMI according to food allergic status	record of everything eaten and drunk over four consecutive days, including one weekend day. Food allergic adolescents consumed a significantly lower percentage energy from fat but higher percentage energy from carbohydrate than control participants	there was a greater proportion of males in the food allergy adolescent group
	one of the following foods: egg, milk, peanuts, tree nuts, sesame, crustaceans, fish, or wheat adolescents with FAs * (n = 50) medium age 14.5 (SD 2.4) adults with FAs * (n = 23) medium age 39.4 (SD 13.7) * FA: peanut, tree nut, fruits, egg, milk, and sesame	adolescents without FAs (n = 33) medium age 14.5 (2.2) adults without FAs (n = 47) medium age 30.2 (SD 9.5)		adolescents with food allergy had higher intakes of niacin and selenium than adolescents without (*p* < 0.05). This difference persisted when dietary supplements were removed from the analysis across all participants, the intake of several micronutrients was suboptimal. There was no difference in protein or energy intake, according to food allergic status.	the food-allergic participants recruited were predominantly members of a support charity, while the majority of control participants were recruited through advertisements on university websites a substantial proportion of all participants did not meet RNIs for micronutrients, particularly minerals, which may indicate a poor choice of foods with low nutrient density

Other commonly inadequate nutrients included calcium and vitamin D; both these deficiencies have been observed in children avoiding dairy products [8]. Other micronutrient deficiencies, including zinc, iodine, vitamin B12, and folate, have also been observed in this group [14,16,17].

### 3.2. Impact of Multiple Pediatric FAs on Growth Parameters

All the studies evaluated growth parameters in relation to dietetic treatment, with two of them reporting longitudinal observations. Growth measures included auxological variables and weight-for-age, height-for-age, head circumference-for-age, BMI-for-age, and weight-for-height Z-scores, and height-for-age percentiles.

Four studies reported that children with multiple FAs have lower growth parameters compared to their non-allergic peers [7,9,14,16]. The severity and duration of food restrictions play a critical role in determining growth outcomes.

Longitudinal data suggest that children with multiple FAs who avoid multiple major food groups (such as dairy, wheat, and soy) are at higher risk of short stature due to chronic nutrient deficiencies and suboptimal caloric intake [7].

## 4. Discussion

Our systematic review confirms a significant impact of pediatric FAs in the nutritional adequacy and nutritional parameters of affected children.

Findings on nutrient intake in children with FAs, although difficult to synthesize due to the wide variation in reference standards and definitions used across studies, confirm the possibility of multiple nutritional deficiencies in children with multiple FAs.

Dietary counseling emerges as a central factor in mitigating nutritional risks. In a longitudinal study, children with FAs who received counseling significantly improved their intake of energy, protein, calcium, and zinc over a six-month period [7]. Conversely, children without counseling were more likely to fall below critical thresholds for the recommended daily intake of calcium and vitamin D, reinforcing the idea that professional guidance is not merely helpful, but essential for maintaining adequate nutritional status in this population. Calcium and vitamin D deficiencies remain the most frequently reported concerns, particularly in children with CMA. Inadequate intake was more common in those who did not receive dietary counseling, emphasizing the central role of professional guidance in preventing and treating micronutrient deficiencies. Importantly, studies conducted prior to the widespread use of amino-acid-based formulas may overestimate current risks, highlighting the need for a context-aware interpretation of older data [18]. Vitamin E intake, on the other hand, was reported to be higher among allergic children in some studies, possibly due to the oil supplementation prescribed during nutritional counseling to increase energy and fat intake [19]. Some studies found higher intakes of niacin and selenium among allergic children compared to controls. This may be partially explained by dietary substitutions such as soy-based products, which are commonly used in milk-free diets and naturally contain higher levels of selenium. These discrepancies may stem from varying counseling practices or socioeconomic factors, but they also highlight the heterogeneity of dietary responses within the allergic pediatric population [17]. In addition to micronutrient deficiencies, imbalances in macronutrient distribution have also been noted. While total energy intake may be similar between allergic and non-allergic children, differences in macronutrient sources suggest possible dietary adaptations. Maslin et al. found that adolescents with FAs derived a lower percentage of their energy from fat and a higher proportion from carbohydrates, possibly due to the avoidance of high-fat processed foods that commonly contain allergens [17]. While this shift may reduce exposure risk, it may also have unintended nutritional consequences, particularly if fat-soluble vitamins or essential fatty acids are insufficiently replaced.

Essential fatty acid deficiencies, although less frequently studied, are another area of concern. Given the exclusion of key sources of *n*-3 polyunsaturated fatty acids in children with multiple FAs, there may be long-term consequences for neurodevelopment, cardiovascular health, and immune function. These findings point to the need for targeted dietary strategies or supplementation to prevent cumulative functional deficits.

Of particular note is the recent shift in focus from individual nutrient intake to overall dietary diversity. Although still underexplored, initial findings suggest that children with FAs may not necessarily have lower diet diversity than healthy controls [14]. This counters the common assumption that FA invariably leads to dietary monotony and underscores the adaptability of well-supported families in maintaining varied diets despite restrictions.

In terms of growth outcomes, evidence from the studies reviewed suggests that children with multiple FAs may be at increased risk of growth impairments and nutritional inadequacies. While only a minority of studies (4 out of 11) explicitly reported abnormal growth patterns in children with multiple FAs, these findings are significant in light of the heterogeneity in the study design, population characteristics, and definitions of malnutrition. Growth impairment appears to be more pronounced in children with three or more FAs, pointing toward a possible cumulative effect of dietary restrictions on nutritional status, resulting in malnutrition. However, methodological factors may influence the reported estimates. For instance, the higher malnutrition rates reported by Flammarion et al. [9]. could be attributed to their stricter inclusion criteria, excluding children who had only recently (less than four months) begun dietary avoidance.

Some studies underscore the potential benefits of targeted nutritional interventions. The work by Berni Canani et al. highlights that tailored dietary counseling can effectively reverse malnutrition, bringing anthropometric parameters back in line with those of healthy peers in promoting adequate growth and maintaining healthy anthropometric outcomes in children with FAs [7].

Interestingly, recent studies challenge the traditional view that children with FAs are uniformly at risk of undernutrition. In Dziechciarz et al., a subset of children with CMA was found to be at risk of overweight, a prevalence comparable to that of the general pediatric population [15]. These data suggest that exclusion diets do not necessarily protect against excess caloric intake and may, in some cases, favor the consumption of energy-dense but nutrient-poor alternatives. This dual risk of both undernutrition and overnutrition reflects the complexity of dietary management in this population. One limitation of the study by Dziechciarz et al. is that the sample included only children with suspected or confirmed CMA, with only half of them also having reported allergies to additional foods [15].

In Papachristou et al. [14], children with FAs exhibited a lower BMI and higher rates of underweight, despite a higher reported energy intake. This apparent paradox raises questions about nutrient absorption and utilization, pointing to potential roles of chronic inflammation, altered gut permeability, and persistent gastrointestinal symptoms. Yet, as with many cross-sectional studies, these associations remain speculative and lack the evaluation of growth trajectories over time.

Notably, not all findings align. Mehta et al. [16] found no differences in the growth parameters between children with and without FAs. The authors attribute this to their broader recruitment from a general pediatric population, in contrast to tertiary allergy clinics in previous studies that may overrepresent more severe phenotypes. The influence of socioeconomic status also emerged, with divergent growth patterns seen between children with private versus public insurance.

Among allergen-specific effects, CMA appears most strongly associated with impaired growth, particularly beyond infancy. This may reflect the nutritional contribution of breast milk or formula in the first two years of life, which may mask deficiencies in early stages. By contrast, avoidance of other allergens such as egg, peanut, or fish was not consistently associated with growth impairment, suggesting that the nutritional risks are not uniform across different dietary exclusions.

Finally, data on adolescents remain limited, with at least one study reporting no association between food allergy status and BMI in older children [17]. Whether this reflects true normalization of growth over time or simply reflects the insensitivity of BMI as a late-stage indicator remains an open question.

Taken together, these findings suggest that growth impairment is a concern in a subset of children with multiple FAs, but its manifestation is variable and context-dependent. The evidence underscores the need for individualized, age-specific, and allergen-specific nutritional monitoring, and calls for longitudinal studies that can better capture the dynamics of growth and nutrition across the pediatric years.

This systematic review presents several strengths. It is one of the few to specifically focus on children with multiple FAs, rather than isolated food allergies, providing a more comprehensive overview of the associated nutritional and growth-related challenges. Second, the use of clearly defined inclusion criteria, a systematic literature search according to PRISMA guidelines, and a critical appraisal of study quality contributes to the methodological robustness and transparency of the review. However, some limitations should be acknowledged. The methodological variability across studies, including diagnostic criteria for food allergies, age ranges of the studied populations, and definitions of nutritional and growth outcomes, may reduce the overall comparability of findings and limit the strength and generalizability of the evidence synthesized. Additionally, some of the included studies lacked standardized comparisons with dietary reference values or longitudinal follow-up, making it difficult to assess nutritional adequacy and growth trends over time. Moreover, reliance on parent-reported dietary data may have introduced recall and reporting bias.

In summary, the evidence indicates that while children with multiple FAs are at risk of nutritional imbalances, these risks are neither uniform nor inevitable. Nutritional counseling plays a pivotal role in mitigating deficiencies and optimizing dietary patterns. Prioritizing standardized methods for nutrient assessment, including measures of diet quality and diversity, and investigating the long-term effects of allergen avoidance on nutrient status and functional outcomes would be highly valuable for advancing clinical practice and research.

## 5. Conclusions

To fully elucidate the impact of food avoidance, future research should focus on specific patterns of dietary elimination and their associations with nutritional deficiencies and growth outcomes. Further measures of growth, such as body composition, could better clarify the impact of FAs on growth parameters. Clinicians should ensure a regular dietetic follow-up and routine monitoring of nutrient intake and growth parameters in children with multiple FAs, with particular attention to calcium, vitamin D, protein, and essential fatty acid intake. Early identification of nutritional risks and individualized dietary counseling are essential to prevent long-term deficiencies and support optimal growth. Longitudinal study designs are essential and should account for key variables such as socioeconomic status, dietary adherence, and diet diversity. Moreover, the evaluation of personalized nutritional interventions, including the use of fortified foods and dietary supplements, alongside the investigation of emerging therapies such as oral immunotherapy, may contribute to the development of comprehensive, multidisciplinary strategies aimed at optimizing clinical management in this vulnerable population.

## Abbreviations

The following abbreviations are used in this manuscript:

FAs	Food allergies
EFSA	European Food Safety Authority
PRISMA	Preferred Reporting Items for Systematic Reviews and Meta-Analysis
JBI	Joanna Briggs Institute
CMA	Cow’s milk allergy
CMP	Cow’s Milk Protein
CDC	Centers for Disease Control and Prevention
DHA	docosahexanoic acid
DPA	docosapentaenoic acid
DRI	dietary reference intake
EFA	essential fatty acid
NCHS	National Center for Health Statistics
RDA	recommended daily allowance
RDI	reference daily intake
WNL	within normal limits
